# GSK3α/β Restrain IFN-γ–Inducible Costimulatory Molecule Expression in Alveolar Macrophages, Limiting CD4^+^ T Cell Activation

**DOI:** 10.4049/immunohorizons.2300107

**Published:** 2024-02-12

**Authors:** Laurisa M. Ankley, Kayla N. Conner, Taryn E. Vielma, Jared J. Godfrey, Mahima Thapa, Andrew J. Olive

**Affiliations:** Department of Microbiology, Genetics, and Immunology, College of Osteopathic Medicine, Michigan State University, East Lansing, MI

## Abstract

Macrophages play a crucial role in eliminating respiratory pathogens. Both pulmonary resident alveolar macrophages (AMs) and recruited macrophages contribute to detecting, responding to, and resolving infections in the lungs. Despite their distinct functions, it remains unclear how these macrophage subsets regulate their responses to infection, including how activation by the cytokine IFN-γ is regulated. This shortcoming prevents the development of therapeutics that effectively target distinct lung macrophage populations without exacerbating inflammation. We aimed to better understand the transcriptional regulation of resting and IFN-γ–activated cells using a new ex vivo model of AMs from mice, fetal liver–derived alveolar-like macrophages (FLAMs), and immortalized bone marrow-derived macrophages. Our findings reveal that IFN-γ robustly activates both macrophage types; however, the profile of activated IFN-γ–stimulated genes varies greatly between these cell types. Notably, FLAMs show limited expression of costimulatory markers essential for T cell activation upon stimulation with only IFN-γ. To understand cell type–specific differences, we examined how the inhibition of the regulatory kinases GSK3α/β alters the IFN-γ response. GSK3α/β controlled distinct IFN-γ responses, and in AM-like cells, we found that GSK3α/β restrained the induction of type I IFN and TNF, thus preventing the robust expression of costimulatory molecules and limiting CD4^+^ T cell activation. Together, these data suggest that the capacity of AMs to respond to IFN-γ is restricted in a GSK3α/β-dependent manner and that IFN-γ responses differ across distinct macrophage populations. These findings lay the groundwork to identify new therapeutic targets that activate protective pulmonary responses without driving deleterious inflammation.

## Introduction

Macrophages are innate immune cells that play an important role in sensing the environment, initiating inflammation, and helping to activate the adaptive immune response (1). In the lungs, both resident and recruited macrophages play important roles in maintaining pulmonary function and protecting against respiratory pathogens (2). Resident lung macrophages, including alveolar macrophages (AMs), reside in the airspace to recycle surfactants produced by the lungs (3). AMs are the first immune cells to detect pathogens in the lungs and are tasked with appropriately responding to stimuli while maintaining pulmonary function (4). During respiratory infections, monocyte-derived inflammatory macrophages are recruited to the lung tissues to support antimicrobial responses and resolve infections (5, 6). Dysregulation of these two important macrophage populations can result in pulmonary dysfunction, susceptibility to infection, and autoinflammatory disease (3, 7, 8). While both resident and recruited macrophages contribute to immune responses in the lungs, their regulation and functional mechanisms are distinct (9). For example, several studies suggest that AMs and recruited macrophages have different abilities to activate protective T cell responses, yet how this process is regulated remains unknown (10–12).

One cue that drives T cell interactions with macrophages is the cytokine IFN-γ. IFN-γ binds to IFN-γR on macrophages, activating Jak/Stat signaling pathways to drive the transcriptional induction of hundreds of genes that are mediated by IFN regulatory factors (13). Among these IFN-γ–inducible genes are important T cell modulatory markers, including Ag presentation machinery, such as MHC class I and II (MHC-I and MHC-II), as well as costimulatory/inhibitory markers, including CD40, CD80, CD86, and PD-L1. In bone marrow–derived macrophages (BMDMs), IFN-γ responses can be further fine-tuned through the activity of key regulators including the kinases GSK3α/β and mTOR (14–17). GSK3α/β are essential kinases that control a range of developmental and regulatory functions, such as WNT signaling, cell death pathways, and inflammation. GSK3α/β are known to be important in neurons and epithelial cells, but their role in macrophages is less appreciated (18). How GSK3α/β control macrophage function and whether the roles of GSK3α/β are conserved in both AMs and recruited macrophages remain unclear (19).

Developing new therapies to combat respiratory infections will require a mechanistic knowledge of regulatory and functional differences between distinct macrophage subsets to ensure effective control of inflammation and T cell effector function in the lungs. However, this understanding requires ex vivo models that faithfully recapitulate in vivo macrophage biology. BMDMs are differentiated from myeloid progenitors and are a widely used model for recruited inflammatory macrophages (20). Following activation with IFN-γ, BMDMs become highly glycolytic, driving inflammatory cytokine production and directly modulating T cell responses in a similar manner to recruited macrophages (14, 21, 22).

Until recently, the availability of ex vivo models for AMs posed a challenge because AMs are notoriously difficult to maintain and isolate (23, 24). Thus, this technical hurdle has limited our understanding of regulatory networks that control AM functionality. It is important to understand how these lung-resident cells uniquely respond to inflammatory signals compared with other macrophages to develop lung-specific therapies that protect against infection while maintaining pulmonary function. To address this gap, several groups developed approaches to culture AM-like cells ex vivo that maintain AM functions (23, 25–27). Although the details of these approaches differ, they all leverage lung-specific cytokine cues from GM-CSF and TGF-β that are required to maintain AM populations in the lung environment. We previously developed an ex vivo AM model known as fetal liver–derived alveolar-like macrophages (FLAMs) that uses fetal liver cells, which are AM progenitors, to interrogate AM function. Our previous work showed that FLAMs maintain high expression of the AM surface marker SiglecF and the key transcription factor Pparγ (23). FLAMs are easy to isolate, culture, and expand, which, along with their genetic tractability, are strengths that allow mechanisms underlying AM functions to be interrogated.

Here, we examined the transcriptional profile of resting and IFN-γ–activated FLAMs and immortalized BMDMs (iBMDMs) to better define functional differences between these key macrophage subsets. Our results show that FLAMs are similar to primary AMs, and although both FLAMs and iBMDMs respond to IFN-γ, they exhibit unique transcriptional profiles. The regulation of these IFN-γ responses is also distinct, with GSK3α/β playing unique roles in FLAMs and iBMDMs. Modulating GSK3α/β activity in IFN-γ–activated FLAMs results in the robust production of type I IFN, which contributes to the induction of costimulatory molecules and increases the capacity of both FLAMs and AMs to activate CD4^+^ T cells. Our results suggest that AMs are restrained in their capacity to activate CD4^+^ T cells following IFN-γ stimulation and that the IFN-γ response is uniquely regulated in different macrophage subsets. These results have implications when considering host-directed therapies that target distinct macrophage populations in the pulmonary tissue.

## Materials and Methods

### Animal experiments

All cell isolation involving live mice was performed in accordance with the recommendations from the Guide for the Care and Use of Laboratory Animals of the National Institutes of Health and the Office of Laboratory Animal Welfare. Mouse studies were performed using protocols approved by the Institutional Animal Care and Use Committee. All mice were housed and bred under specific pathogen-free conditions and in accordance with Michigan State University (PROTO202200127) Institutional Animal Care and Use Committee guidelines. All mice were monitored and weighed regularly. C57BL6/J mice (catalog no. 000664) and *Ifnar1^−/−^* mice (catalog no. 028288) were purchased from The Jackson Laboratory.

### Cell isolation

J2 virus–immortalized Cas9^+^ BMDMs (iBMDMs) were isolated and immortalized from C57BL/6J mice as previously described (14, 18). FLAMs were isolated from C57BL/6J mice or *Ifnar1^−/−^* mice as previously described (23, 25). Briefly, fetal livers were extracted from euthanized dams immediately after sacrifice. Each liver was ground into a single-cell suspension and filtered through a 40-µm mesh screen and plated in one well of a six-well plate in FLAM medium (see below). Primary AMs were isolated by bronchoalveolar lavage of C57BL/J6 mice, as previously described and cultured in FLAM medium (54). P25 TCR-Tg CD4^+^ T cells (41, 42) were isolated from the lymph nodes and spleens of transgenic P25TCR mice kindly shared by Joel Ernst. The spleen and lymph nodes were homogenized, passed through a 70-µm strainer, and washed with RPMI medium (Life Technologies, catalog no. 11875093). CD4^+^ T cells were enriched using a MojoSort T cell isolation kit (BioLegend, catalog no. 480006) following the manufacturer’s protocol.

### Cell culture

iBMDMs were maintained in DMEM (HyClone Cytiva, catalog no. SH30243.01) supplemented with 10% FBS (R&D Systems, catalog no. S11550). iBMDMs were passaged once they reached 70–90% confluency. The cells were used in experiments after 1 week of culture. FLAMs were maintained in RPMI 1640 complete medium supplemented with 10% FBS, 20 ng/ml recombinant human TGF-β1 (PeproTech, catalog no. 100-21), and 30 ng/ml recombinant murine GM-CSF (PeproTech, catalog no. 300-03). FLAM medium was refreshed every 3 d. FLAMs were passaged at 70–90% confluency. Primary AMs were cultured in complete RPMI 1640 supplemented with 10% FBS, 30 ng/ml GM-CSF, and 20 ng/ml recombinant human TGF-β1. CD4^+^ T cells were cultured in complete RPMI 1640 medium supplemented with 10% FBS and penicillin–streptomycin (50 U/ml penicillin, 50 mg/ml streptomycin) (Life Technologies, catalog no. 15140-122). All cells were incubated in 5% CO_2_ at 37°C.

### Macrophage treatment conditions

For all assays, unless otherwise indicated, FLAMs and iBMDMs were plated at a density of 1 × 10^6^ cells per well in 6-well tissue culture plates and were allowed to adhere overnight. The following day, the cells were treated with DMSO (Fisher Chemical, catalog no. D128500), DMSO, and 6.25 ng/ml IFN-γ (PeproTech, catalog no. 315-05), 10 μM CHIR99021 (Sigma-Aldrich, catalog no. SML1046), or both 10 μM CHIR99021 and 6.25 ng/ml IFN-γ for 24 h. In experiments with TNF (PeproTech, catalog no. 31501A) or IFN-β (BioLegend, catalog no. 581304), recombinant cytokines were added at 20 ng/ml for 24 h. For the TNF receptor (TNFR) blocking experiment, an anti-TNFR blocking Ab (BioLegend, catalog no. 113104) was added at 1.25 ng/ml 24 h prior to IFN-γ activation.

### Flow cytometry

For all experiments, the cells were lifted by gentle scraping, washed with PBS, and stained with MHC-II-FITC (BioLegend, catalog no. 107606), CD40-APC (BioLegend, catalog no. 124612), PDL1-BV421 (BioLegend, catalog no. 124315), CD80-PE (BioLegend, catalog no. 104708), CD86-APC-Cy7 (BioLegend, catalog no. 104708), TLR2-APC (BioLegend, catalog no. 153006), MRC1-PE (BioLegend, catalog no. 141706), Siglec1-FITC (BioLegend, catalog no. 142406), CD14-PE-Cy7 (BioLegend, catalog no. 123316), CD11a-PE (BioLegend, catalog no. 153103), and CD69-PE (BioLegend, catalog no. 104508) (all diluted 1:400 in PBS). The cells were then washed three times in PBS and fixed with 1% formaldehyde (J. T. Baker, catalog no. JTB-2106-01) in PBS. Flow cytometry was performed on a BD LSR II or an Attune CytPix at the Michigan State University Flow Cytometry Core, and the data were analyzed using FlowJo (version 10.8.1).

### Cytokine profiling

FLAMs and iBMDMs were treated as described above for 24 h, and supernatants were collected for cytokine profiling by Eve Technologies using a mouse cytokine/chemokine 31-Plex discovery assay array. For TNF (BioLegend ELISA Max mouse TNF-α, catalog no. 430901) or IFN-β1 (Invivogen murine IFN-β bioluminescent ELISA kit 2.0, catalog no. luex-mifnbv2) ELISAs using supernatants from primary AMs, the manufacturer’s instructions were followed.

### RNA sequencing and analysis

FLAMs and iBMDMs were plated in 6-well plates at 1 × 10^6^ cells/well and treated with IFN-γ and CHIR99021 as described above for 24 h. The Direct-zol RNA extraction kit (Zymo Research, catalog no. R2072) was used to extract RNA according to the manufacturer’s protocol. Quality was assessed by the Michigan State University Genomics Core using an Agilent 4200 TapeStation system. Libraries were prepared using an Illumina stranded mRNA library prep kit (Illumina, catalog no. 20040534) with IDT for Illumina RNA Unique Dual Index adapters following the manufacturer’s recommendations, except that half-volume reactions were performed. The generated libraries were quantified and assessed for quality using a combination of Qubit dsDNA HS (Thermo Fisher Scientific, catalog no. Q32851) and Agilent 4200 TapeStation HS DNA1000 assays (Agilent, catalog no. 5067-5584). The libraries were pooled in equimolar amounts, and the pooled library was quantified using an Invitrogen Collibri quantification quantitative PCR kit (Invitrogen, catalog no. A38524100). The pooled library was loaded onto two lanes of a NovaSeq S1 flow cell, and sequencing was performed in a 1 × 100-bp single-read format using a NovaSeq 6,000 v1.5 100-cycle reagent kit (Illumina, catalog no. 20028316). Base calling was performed with Illumina real-time analysis (version 3.4.4), and the output of real-time analysis was demultiplexed and converted to the FastQ format with Illumina Bcl2fastq (version 2.20.0).

All RNA sequencing (RNA-seq) analyses were performed using the Michigan State University High Performance Computing Center. Read quality was assessed using FastQC (version 0.11.7) (55). Read mapping was performed against the GRCm39 mouse reference genome using Bowtie2 (version 2.4.1) (56) with default settings. Aligned read counts were assessed using the FeatureCounts function from the Subread package (version 2.0.0) (57). Differential gene expression analysis was conducted using the DESeq2 package (version 1.36.0) (58) in R (version 4.2.1). One IFN-γ–treated FLAM sample did not pass quality control and was not included in the analysis. All raw sequencing data, raw read counts, and normalized read counts are available through the NCBI Gene Expression Omnibus (GSE239280, https://www.ncbi.nlm.nih.gov/geo/query/acc.cgi?acc=GSE239280).

Core AM upregulated signature genes were compared between FLAMs and iBMDMs from our study and AMs and peritoneal macrophages from ImmGen (GSE122108 https://www.ncbi.nlm.nih.gov/geo/query/acc.cgi?acc=GSE122108) (30). Raw counts were compiled and normalized in DESeq2. A box plot was generated in GraphPad Prism using normalized counts for core AM upregulated signature genes. Gene set enrichment analysis (GSEA) was used to identify enriched pathways in the RNA-seq dataset. Genes in the indicated comparisons were ranked using DeSeq2, and the “GSEA preranked” function was used to complete functional enrichment using default settings for hallmark pathways from mice. We acknowledge our use of the gene set enrichment analysis, GSEA software, and Molecular Signature Database (MSigDB) (59) (http://www.broad.mit.edu/gsea/).

### T cell assays

A previously established coculture system to assess Ag-specific T cell activation was used (20). In short, CD4^+^ T cells were stimulated with p25 peptide-pulsed (sequence: FQDAYNAAGGHNAVF from Genescript) iBMDMs or FLAMs that were irradiated with mitomycin (25 µg/ml) (VWR, catalog no. TCM2320) and had been pretreated with DMSO, IFN-γ, CHIR99021, and CHIR99021/IFN-γ, as described above. Cocultures were supplemented with 10 ng/ml IL-12 (Peprotech, catalog no. 210-12) and 10 µg/ml anti-IL-4 (BioLegend, catalog no. 504-102) to achieve Th1 polarization. Three days after coculture initiation, the surface expression of CD69 was quantified on p25 cells using flow cytometry as described above, and supernatants were used to quantify T cell activation levels with an ELISA (BioLegend, catalog no. 430801) following the manufacturer’s protocols.

### Statistical analysis and data visualization

Statistical analysis was performed using Prism version 10 (GraphPad) as indicated in the figure legends. The data are presented, unless otherwise indicated, as means ± SD. For parametric data, one-way or two-way ANOVA followed by Tukey’s post hoc test was used to identify significant differences between multiple groups, and Student *t* tests were used to compare two groups. For nonparametric data, two-way ANOVAs and Mann–Whitney *U* tests were used to compare multiple groups and two groups, respectively.

## Results

### FLAMs are phenotypically similar to AMs and distinct from BMDMs

**FIGURE 1.**
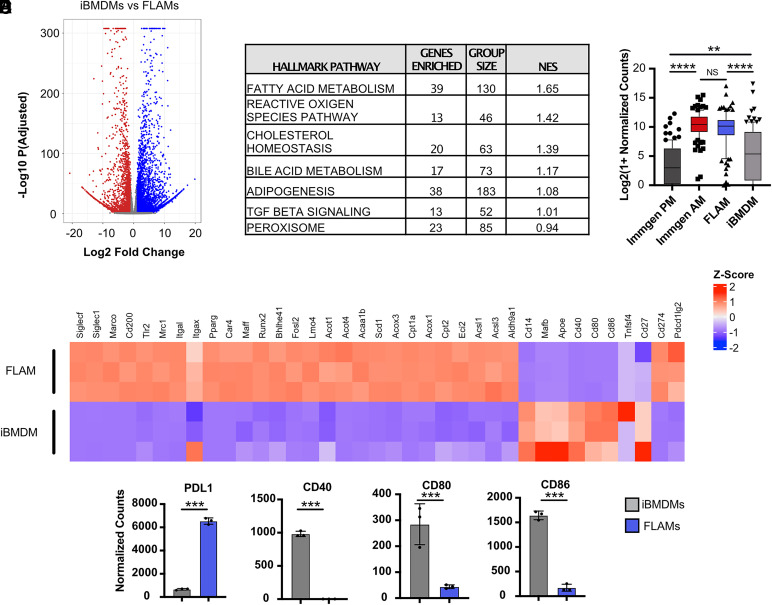
FLAMs are genetically similar to AMs. (**A**) Differentially expressed genes in untreated FLAMs (blue) and iBMDMs (red) were identified using RNA-seq. (**B**) Top seven hallmark pathways enriched in untreated FLAMs. (**C**) Normalized counts of core AM genes were compared among iBMDMs, FLAMs, and previously published datasets from ImmGen (ImmGen PM and ImmGen AM; accession GSE122108, https://www.ncbi.nlm.nih.gov/geo/query/acc.cgi?acc=GSE122108). The box plot shows the median with quartiles representing the 10th to 90th percentile range of the data within that cell type. Each point represents the mean normalized counts of an individual gene. The Mann–Whitney *U* test was used to make statistical comparisons between each cell type and to compare medians. (**D**) Relative expression of genes previously associated with recruited macrophages was compared between FLAMs and iBMDMs and is expressed as a heat map. The color scale represents the Z score calculated from normalized read counts across samples for each gene. (**E**) Normalized counts of costimulatory molecules between untreated FLAMs (blue) and iBMDMs (gray). Adjusted *p* values were determined using DeSeq2. All data points each represent one biological replicate ± the SD from one experiment. ****p < 0.0001, ***p < 0.001, **p < 0.01, *p < 0.05. PM, peritoneal macrophage.

To gain a global understanding of FLAM transcriptional patterns and to identify similarities and differences between FLAMs and other macrophage populations, we conducted RNA-seq analysis of resting FLAMs and iBMDMs. Using differential expression analysis, we identified hundreds of genes that were significantly different between FLAMs and iBMDMs (Fig. 1A, Supplemental Table I). These data suggest that FLAMs and iBMDMs are transcriptionally distinct macrophage populations.

To identify global pathways that were uniquely enriched in FLAMs, GSEA was performed using a ranked gene list generated from the differential expression analysis. Among the top hallmark pathways enriched in FLAMs, we identified fatty acid metabolism, TGF-β signaling, cholesterol homeostasis, and peroxisome pathways (Fig. 1B, Supplemental Table II). Given that AMs drive lipid metabolism in a fashion that is dependent on the transcription factor Pparγ, these data suggest the FLAM transcriptional profile is similar to that of primary AMs (28). To directly evaluate similarity to primary macrophages, we compared the FLAM and iBMDM RNA-seq transcriptional profiles with previously published datasets examining primary AMs and peritoneal macrophages made available through the Immunological Genome Project (ImmGen) (29, 30). In line with the GSEA results, we found that FLAMs were more similar to AMs, whereas iBMDMs were more similar to peritoneal macrophages (Fig. 1C, Supplemental Table III). These findings suggest that FLAMs are a more useful model than BMDMs to study AM-specific functions ex vivo.

To further examine similarities with primary macrophages, we studied a subset of genes that were previously associated with recruited macrophages or AMs (31). We found that iBMDMs expressed high levels of genes associated with recruited macrophages, including CD14, ApoE, and the key transcription factor MafB (Fig. 1D, Supplemental Table IV). In contrast, FLAMs expressed high levels of transcription factors associated with resident lung macrophages, such as Pparγ, Car4, Maff, Fosl2, Bhlhe41, and Runx2. In addition, FLAMs expressed high levels of resident macrophage-associated surface markers, including SiglecF, Siglec1, Marco, CD200, TLR2, MRC1, Itgal, and Itgax, which were expressed at low levels or not expressed in iBMDMs. In line with there being functional similarities between AMs and FLAMs, we observed high expression of genes associated with lipid and cholesterol metabolism in FLAMs (26). Interestingly, when we examined genes that modulate T cell activation, we observed high expression of the coinhibitory markers PDL1 and PDL2 on FLAMs (Fig. 1D, 1E) (32). In contrast, we observed very low expression of costimulatory molecules, including CD40, CD80, and CD86 (Fig. 1D, 1E). These data show that FLAMs express core AM-associated genes, unlike iBMDMs.

**FIGURE 2.**
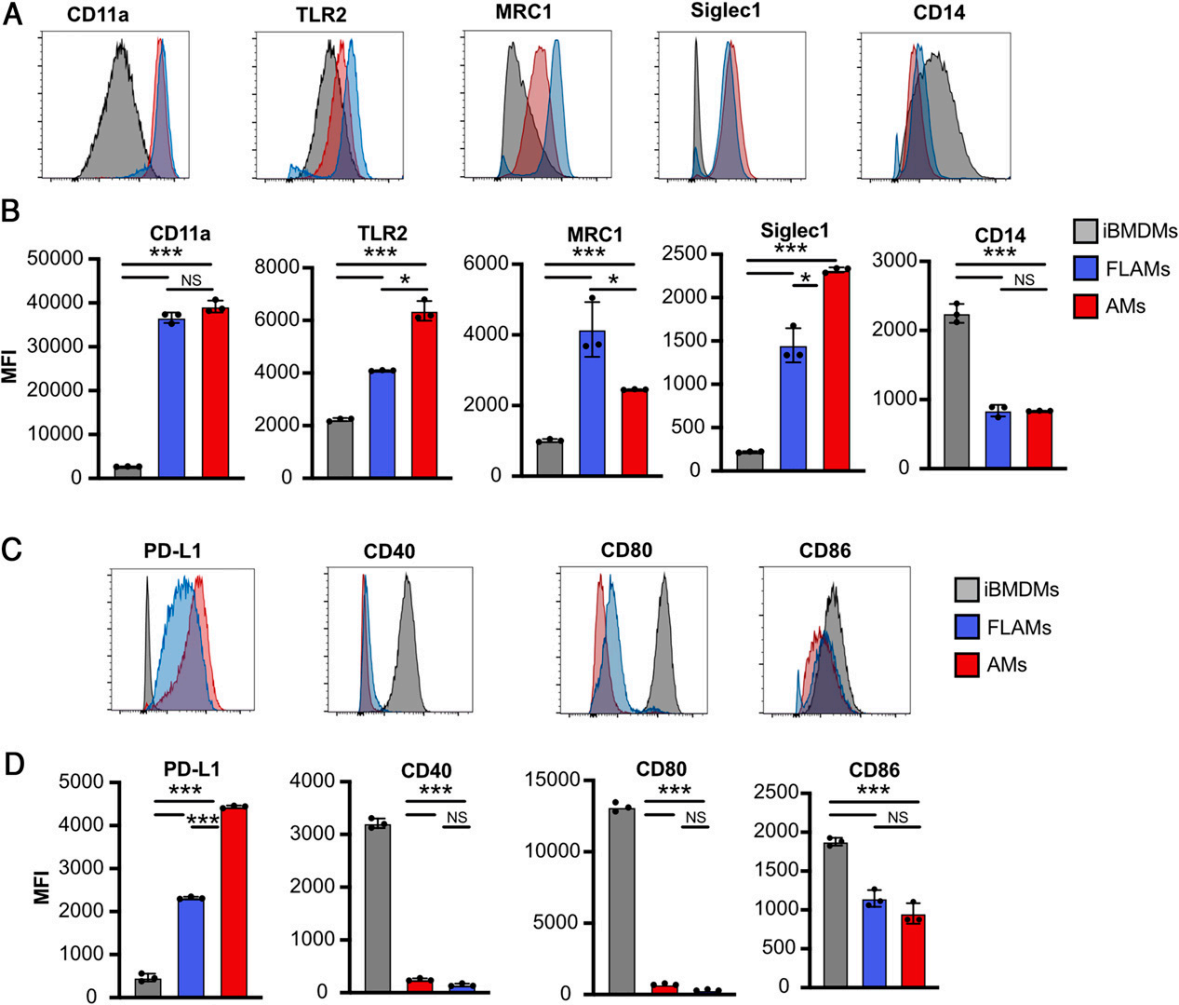
FLAMs express surface markers associated with AMs. (**A**) Representative histograms were overlaid for selected surface markers expressed on AMs (red), FLAMs (blue), or iBMDMs (gray). (**B**) The mean fluorescence intensity (MFI) of each surface marker on each cell type was quantified. One-way ANOVA with a Tukey test for multiple comparisons was used. (**C**) Representative histograms were overlaid for selected costimulatory markers expressed on AMs (red), FLAMs (blue), or iBMDMs (gray). (**D**) The MFI of each costimulatory marker was quantified. One-way ANOVA with a Tukey test for multiple comparisons was used. Each data point represents one biological replicate ± the SD from one experiment of three. ****p < 0.0001, ***p < 0.001, **p < 0.01, *p < 0.05.

To confirm our transcriptional results with an orthologous method, we compared the expression of surface markers predicted to be differentially expressed on AMs and FLAMs relative to iBMDMs with flow cytometry. We found that the surface markers CD11a, TLR2, MRC1, and Siglec1 were all highly expressed on both resting FLAMs and primary AMs, while resting iBMDMs expressed higher levels of CD14 (Fig. 2A, 2B). In agreement with our transcriptional profiling, we also found low expression of costimulatory markers on FLAMs and AMs compared with iBMDMs but high expression of the coinhibitory marker PD-L1 on FLAMs and AMs (Fig. 2C, 2D). Taken together, these results show that FLAMs are transcriptionally distinct from iBMDMs and express several markers associated with primary AMs. Additionally, in resting conditions, FLAMs and AMs express low levels of T cell–activating costimulatory markers.

### IFN-γ induces distinct transcriptional profiles in FLAMs and does not broadly induce T cell costimulatory molecules in FLAMS

**FIGURE 3.**
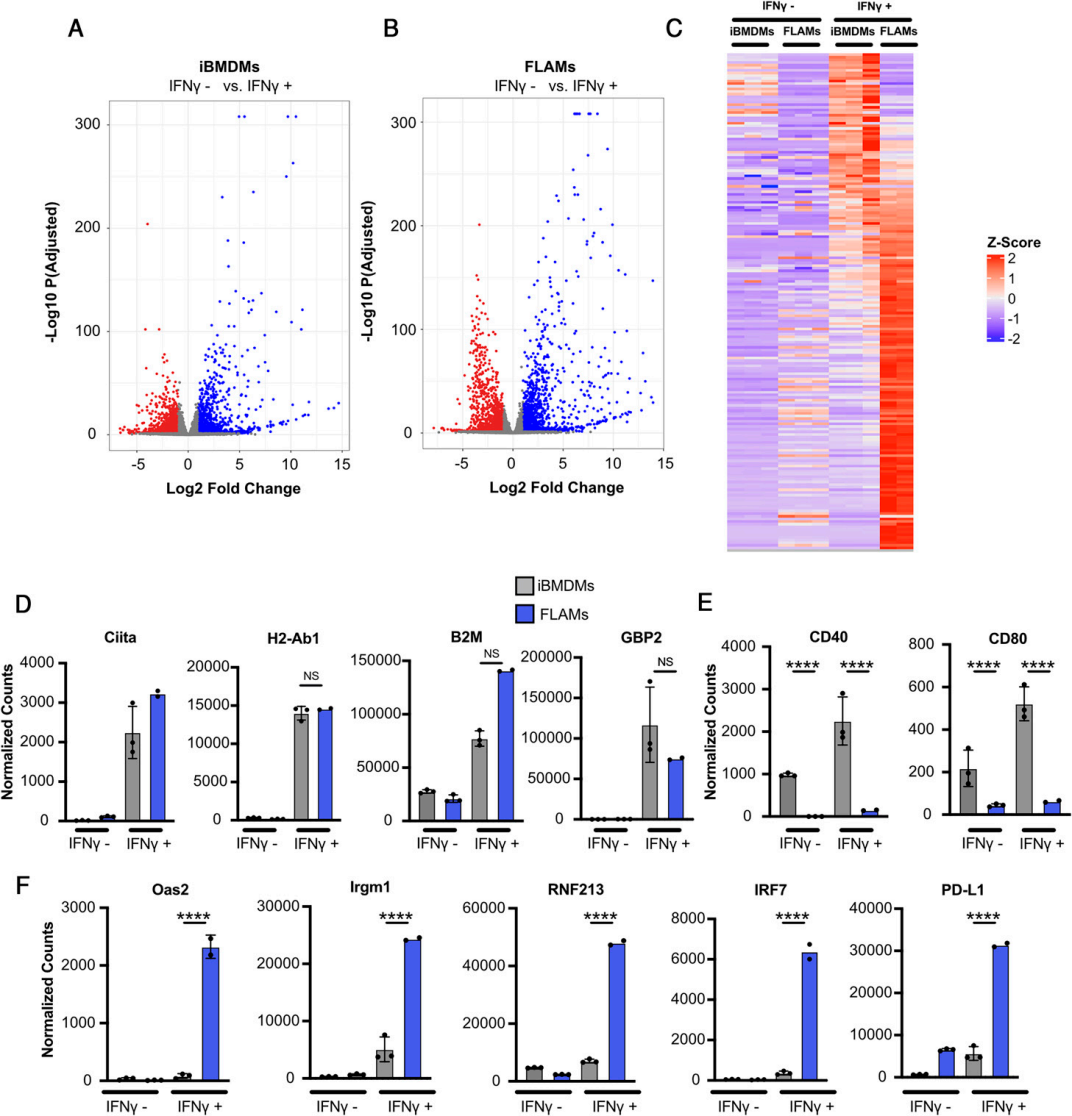
iBMDMs and FLAMs respond differently to IFN-γ stimulation. (**A**) Differential expression of untreated (red) and IFN-γ–stimulated (6.25 ng/ml) (blue) iBMDMs. Colored symbols have an adjusted *p* value < 0.05 and a fold change greater than 2. (**B**) Differential expression of untreated (red) and IFN-γ–stimulated (blue) FLAMs. Colored symbols have an adjusted *p* value < 0.05 and a fold change greater than 2. (**C**) Expression of a subset of ISGs was compared in untreated and IFN-γ–stimulated iBMDMs and FLAMs and is expressed as a heat map. The color scale represents the Z score calculated from normalized read counts across samples for each gene. (**D**) Normalized counts of ISGs that were differentially regulated in iBMDMs (gray) and FLAMs (blue) with and without IFN-γ. Statistical significance was determined based on adjusted *p* values using DeSeq2. The data points each represent one biological replicate from one experiment. (**E**) Normalized counts of costimulatory molecules that were differentially regulated in iBMDMs (gray) and FLAMs (blue) in untreated and IFN-γ–stimulated conditions. (**F**) Normalized counts of cell autonomous restriction factors that were differentially regulated in iBMDMs (gray) and FLAMs (blue) in untreated and IFN-γ–stimulated conditions. Normalized count data points each represent one biological replicate from one experiment ± the SD. MFI data points each represent one biological replicate ± the SD from one representative experiment of three. ****p < 0.0001, ***p < 0.001, **p < 0.01, *p < 0.05.

The cytokine IFN-γ acts as an important regulator of the host response in macrophages by inducing the expression of antimicrobial molecules and T cell modulatory molecules to help drive protective immune responses (33–35). Since transcriptional differences are observed between FLAMs and iBMDMs at baseline, we wondered whether IFN-γ responses would be similar or distinct in these two cell types. To address this question, we conducted global RNA-seq analysis of FLAMs and iBMDMs following IFN-γ activation for 24 h. We first used differential expression analysis to compare IFN-γ–activated FLAMs or iBMDMs to their resting counterparts that were described above. For both iBMDMs and FLAMs, IFN-γ stimulation resulted in the induction of hundreds of genes (Fig. 3A, 3B, Supplemental Table I). This finding suggests that IFN-γ robustly activates both iBMDMs and FLAMs.

To directly compare the IFN-γ–mediated responses of iBMDMs and FLAMs, we visualized the Z score of normalized reads across samples for genes associated with a curated IFN-γ–stimulated gene (ISG) set based on the hallmark pathway for IFN-γ–activated cells (Fig. 3C, Supplemental Table IV). Ag presentation machinery for MHC-I and MHC-II was robustly induced following activation of both FLAMs and iBMDMs (Fig. 3D). However, not all ISGs were differentially induced in stimulated FLAMs and iBMDMs. For example, the costimulatory molecules CD40 and CD80 were robustly induced in iBMDMs, but their expression remained low in FLAMs (Fig. 3E). In contrast, we noted cell-autonomous restriction factors, including *Oas2*, *Irgm1*, and *Rnf213*, were induced at levels over 10-fold higher in FLAMS than iBMDMs (Fig. 3F). Additionally, the transcription factor Irf7 was induced at levels 2–4-fold higher than baseline in iBMDMs following IFN-γ activation, whereas in FLAMs, the observed induction was over 100-fold higher than at baseline. In line with our observations in resting cells, we found that the expression of the coinhibitory marker PDL11 remained over 10-fold higher following IFN-γ activation in FLAMs than in iBMDMs. Taken together, these results show that while both FLAMs and iBMDMs robustly respond to IFN-γ activation, this activation induces distinct transcriptional changes in both cell types, including differences in T cell costimulatory molecules that remained expressed at low levels in FLAMs.

### GSK3α/β inhibition during IFN-γ activation of AMs and FLAMs results in the robust upregulation of costimulatory molecules

**FIGURE 4.**
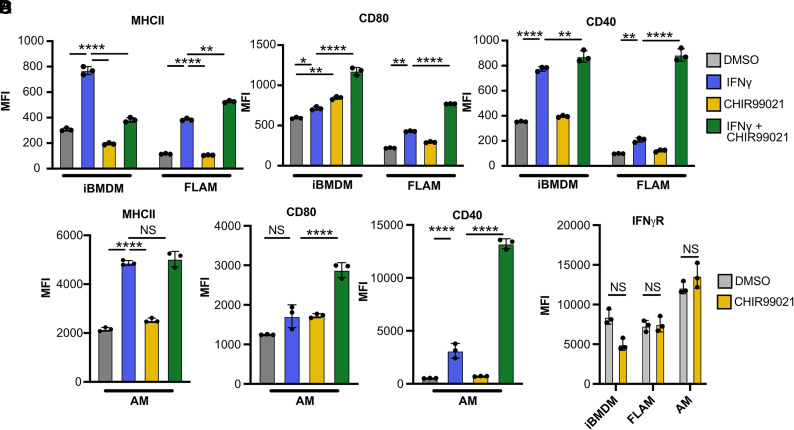
Blocking GSK3α/β during IFN-γ activation of FLAMs drives costimulatory marker expression. iBMDMs and FLAMs were treated with DMSO (gray), DMSO and IFN-γ (6.25 ng/ml) (blue), CHIR99021 (10 μM) (yellow), or CHIR99021 and IFN-γ (10 μM and 6.25 ng/ml) (green) for 24 h. (**A** and **B**) The surface expression of MHC-II (A) and the indicated costimulatory molecules (B) was quantified by flow cytometry. Each data point represents the MFI for each biological replicate ± the SD from one representative experiment of four similar experiments. Statistical significance was determined with two-way ANOVA and a Tukey test for multiple comparisons. (**C**) Primary AMs were treated with DMSO (gray), DMSO and IFN-γ (6.25 ng/ml) (blue), CHIR99021 (10 μM) (yellow), or CHIR99021 and IFN-γ (10 μM and 6.25 ng/ml) (green) for 24 h. The surface expression of the indicated T cell modulatory molecules was quantified by flow cytometry. (**D**) iBMDMs, FLAMs, and AMs were left untreated or treated with CHIR99021 (10 μM) for 24 h, and the surface expression of IFN-γR1 was quantified by flow cytometry. Statistical significance was determined with two-way ANOVA and a Tukey test for multiple comparisons. MFI data points each represent one biological replicate ± the SD from one representative experiment of three. ****p < 0.0001, ***p < 0.001, **p < 0.01, *p < 0.05.

We next examined how the different IFN-γ responses in FLAMs and iBMDMs are regulated. Previous work showed that GSK3α and GSK3β are key regulators that fine-tune the IFN-γ response in iBMDMs and that inhibiting GSK3α/β in iBMDMs blocks a subset of IFN-γ responses, including the expression of the MHC-II transactivator Ciita and subsequent MHC-II expression (14). However, the core IFN-γ signaling pathways, including Stat1 and Irf1, remained intact following GSK3α/β inhibition. We hypothesized that GSK3α/β may contribute to the different IFN-γ responses observed between iBMDMs and AMs. To test this hypothesis, we cultured resting or IFN-γ–activated iBMDMs or FLAMs with and without the highly specific GSK3α/β inhibitor CHIR99021 and then analyzed MHC-II expression with flow cytometry (36). In agreement with our previous results, GSK3α/β blockade in iBMDMs led to a significant reduction in MHC-II on IFN-γ–activated cells (Fig. 4A). In contrast, inhibiting GSK3α/β in IFN-γ–activated FLAMs increased MHC-II expression (Fig. 4A). These data suggest that GSK3α/β have distinct functions in controlling the IFN-γ response in FLAMs and BMDMs.

GSK3α/β were previously shown to modulate costimulatory molecule expression in different cell types (37, 38). Thus, we next tested whether GSK3α/β inhibition alters the IFN-γ–mediated induction of costimulatory molecules. Resting or IFN-γ–activated iBMDMs and FLAMs were treated with DMSO or CHIR99021, and flow cytometry was used to quantify the surface expression of CD40 and CD80. We found that while IFN-γ increased the expression of all markers on iBMDMs, GSK3α/β blockade had no effect on this induction (Fig. 4B). In contrast, while IFN-γ alone resulted in minimal changes to costimulatory molecule expression on FLAMs, GSK3α/β blockade in IFN-γ–activated FLAMs resulted in a robust increase in all costimulatory molecules. These results for MHC-II and costimulatory markers were confirmed in primary AMs (Fig. 4C). It remained possible that CHIR99021 directly altered the expression of IFN-γR on macrophages. To test this possibility, we treated iBMDMs, FLAMs, and AMs with CHIR99021 and examined the surface expression of IFN-γR1 by flow cytometry (Fig. 4D). We observed no significant changes in the expression of IFN-γR1, which suggested that a different mechanism is driving the observed changes in the IFN-γ response. Together, these findings suggest that GSK3α/β play distinct functions in regulating the response to IFN-γ in AMs and BMDMs.

### GSK3α/β inhibition during IFN-γ activation robustly alters the transcriptional landscape of FLAMs

**FIGURE 5.**
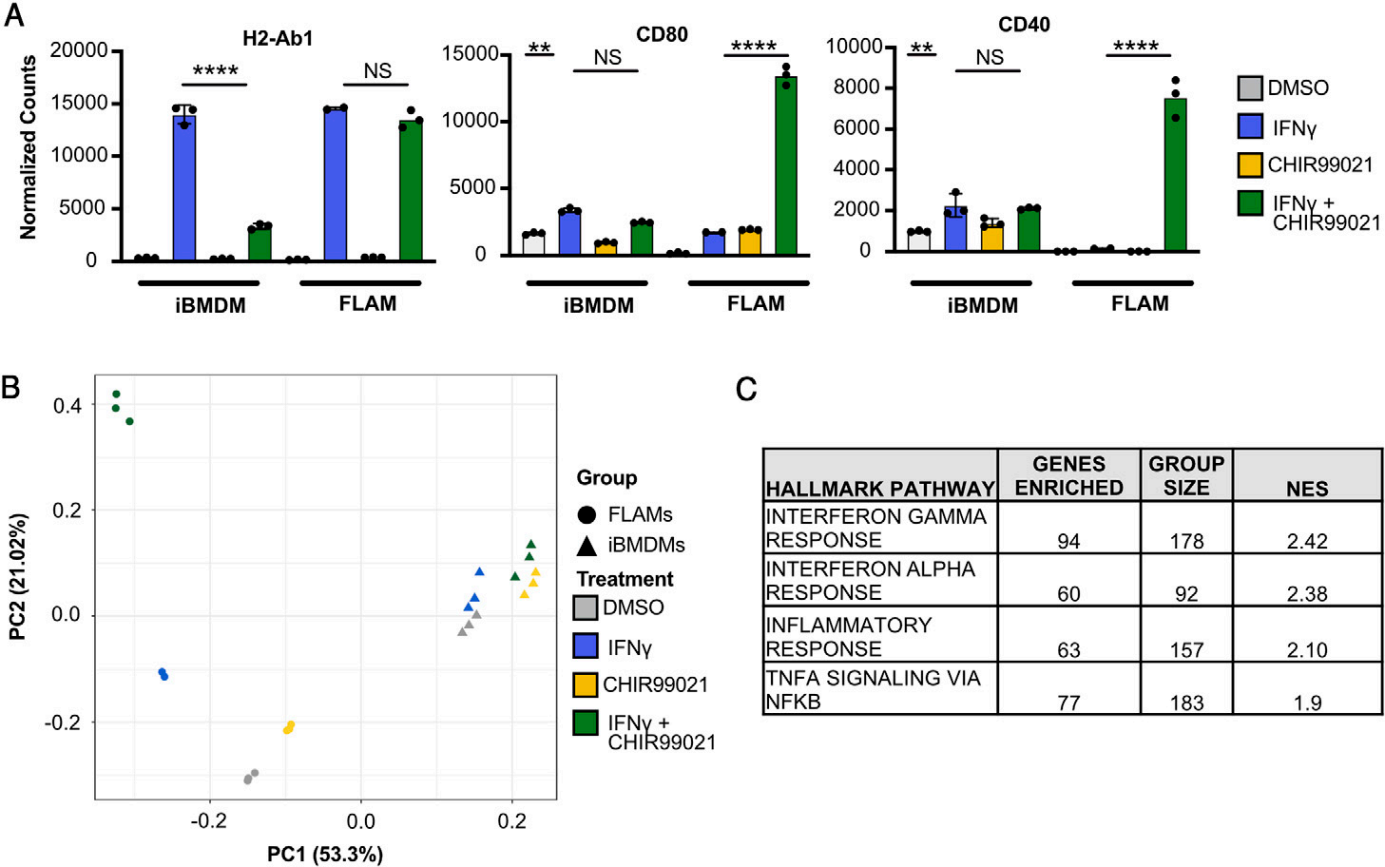
Inhibition of GSK3α/β in IFN-γ–activated FLAMs changes the transcriptional landscape. iBMDMs and FLAMs were treated with DMSO (gray), DMSO and IFN-γ (6.25 ng/ml) (blue), CHIR99021 (10 μM) (yellow), or CHIR99021 and IFN-γ (10 μM and 6.25 ng/ml) (green) for 24 h, and RNA-seq was completed. (**A**) Normalized counts of T cell activation molecules in iBMDMs and FLAMs are shown from RNA-seq analysis in the indicated conditions. Statistical significance was determined with adjusted *p* values calculated by DeSeq2. (**B**) A principal component analysis plot comparing the similarity of iBMDMs and FLAMs in the indicated conditions is shown. (**C**) The top four hallmark pathways enriched using GSEA are shown to compare GSK3α/β-inhibited/IFN-γ–stimulated FLAMs and IFN-γ–stimulated FLAMs from a ranked list calculated by DeSeq2. Normalized count data points each represent one biological replicate ± the SD from one experiment. MFI points each represent one biological replicate ± the SD from one representative experiment of three. ****p < 0.0001, ***p < 0.001, **p < 0.01, *p < 0.05.

Because GSK3α/β inhibition had different impacts on a subset of IFN-γ responses in BMDMs and FLAMs, we next examined global transcriptional changes that occurred during GSK3α/β inhibition. RNA-seq analysis was conducted on resting and IFN-γ–activated iBMDMs and FLAMs in the presence of CHIR99021, and these results were compared with the above RNA-seq analysis in resting and IFN-γ–activated iBMDMs and FLAMs. First, we examined MHC-II and costimulatory marker expression and found that in line with our flow cytometry results, FLAMS treated with combination IFN-γ and CHIR99021 exhibited no change in H2-Ab1 (MHC-II) expression but over 100-fold induction of CD80 and CD40 that was not observed in any other condition (Fig. 5A, Supplemental Table I). Principal component analysis of all eight RNA-seq conditions (± IFN-γ ± CHIR99021 in iBMDMs and FLAMs) revealed clear differences in the transcriptional landscape of iBMDMs and FLAMs (Fig. 5B). All iBMDM samples clustered closely within the principal component analysis plot, with distinct but small shifts in the transcriptomes following IFN-γ and/or GSK3α/β inhibition. Compared to resting iBMDMs, resting FLAMs were greatly shifted along PC1, in line with the above results showing distinct transcriptional landscapes in these resting cell types. While the shifts in the transcriptional profile of FLAMS in response to either IFN-γ activation or GSK3α/β blockade were similar to those observed in iBMDMs, the combination of IFN-γ and CHIR99021 resulted in a major shift in the transcriptional landscape of FLAMs along PC2. These results show that GSK3α/β are key regulators of the IFN-γ response in FLAMs and that the combination of IFN-γ activation and GSK3α/β blockade drives a synergistic transcriptional response not observed in any other FLAM or iBMDM condition.

To understand which pathways are altered during GSK3α/β inhibition in IFN-γ–activated FLAMs, we next used GSEA based on a differential expression ranked list to compare IFN-γ–activated FLAMs in the presence and absence of GSK3α/β inhibition. We found both IFNα and TNF pathways, in addition to IFN-γ, were all significantly enriched in GSK3α/β-inhibited, IFN-γ–activated FLAMs (Fig. 5C, Supplemental Table II). These results suggest that blockade of GSK3α/β during IFN-γ activation of FLAMs drives inflammatory cytokine responses that may contribute to the expression of key IFN-γ–inducible genes, including costimulatory markers.

### Type I IFN and TNF contribute to the upregulation of costimulatory molecules on IFN-γ–activated FLAMs when GSK3α/β are inhibited

**FIGURE 6.**
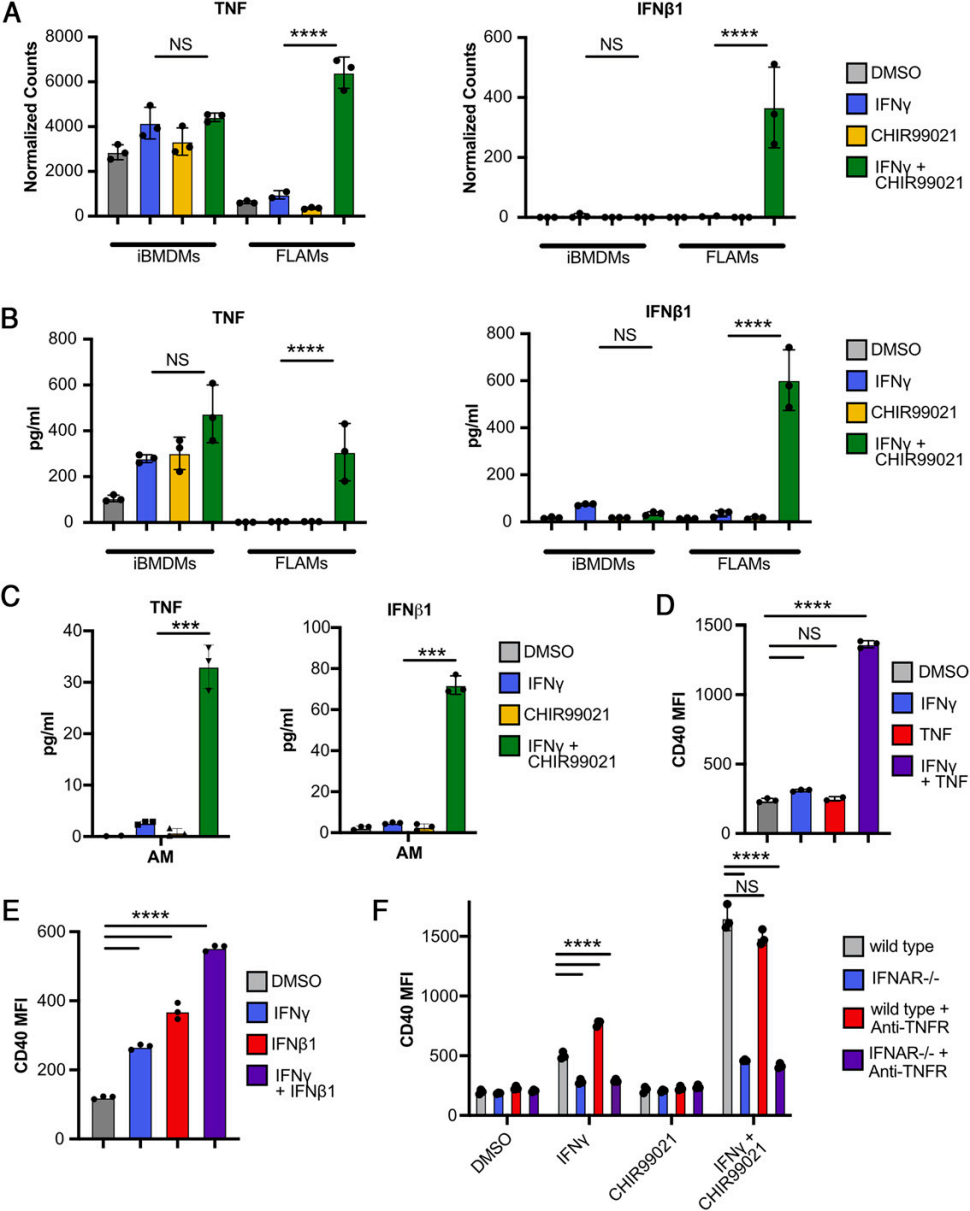
TNF and type I IFN contribute to CD40 expression on IFN-γ–stimulated FLAMs. (**A**) Normalized counts are shown for TNF (*left*) and IFN-β (*right*) from iBMDMs and FLAMs treated with DMSO (gray), DMSO and IFN-γ (6.25 ng/ml) (blue), CHIR99021 (10 μM) (yellow), or CHIR99021 and IFN-γ (10 μM and 6.25 ng/ml) (green) for 24 h. Statistical significance was determined with adjusted *p* values using DeSeq2. (**B**) Production of TNF (*left*) and IFN-β (*right*) from iBMDMs and FLAMs in identical conditions as in (A). Statistical significance was determined with two-way ANOVA and a Tukey test for multiple comparisons. (**C**) The secretion of TNF and IFN-β1 from primary AMs was quantified by ELISA following treatment with DMSO (gray), IFN-γ (6.25 ng/ml; (blue), CHIR99021(10 µM; yellow), or IFN-γ and CHIR99021(6.25 ng/ml and 10 µM, respectively; green) for 24 h. Statistical significance was determined with two-way ANOVA and a Tukey test for multiple comparisons. (**D**) MFI of CD40 for FLAMs treated with DMSO (gray), IFN-γ (6.25 ng/ml) (blue), TNF (20 ng/ml) (red), or IFN-γ and TNF (6.25 ng/ml and 20 ng/ml) (purple) for 24 h. Statistical significance was determined with two-way ANOVA and a Tukey test for multiple comparisons. (**E**) MFI of CD40 for FLAMs treated with DMSO (gray), IFN-γ (6.25 ng/ml) (blue), IFN-β1 (20 ng/ml) (red), or IFN-γ and IFN-β1 (6.25 ng/ml and 20 ng/ml) (purple) for 24 h. Statistical significance was determined with two-way ANOVA and a Tukey test for multiple comparisons. (**F**) MFI of CD40 for WT (gray), IFNAR^−/−^ (blue), WT + anti-TNF (red), and IFNAR^−/−^ + anti-TNF (purple) FLAMs that were cultured with the indicated treatments for 24 h. Statistical significance was determined with two-way ANOVA and a Tukey test for multiple comparisons. Normalized counts points each represent three biological replicates ± the SD from one experiment. MFI and cytokine quantification points each represent one biological replicate ± the SD from one representative experiment of three. ****p < 0.0001, ***p < 0.001, **p < 0.01, *p < 0.05.

We next interrogated the mechanisms driving costimulatory marker induction on GSK3α/β-inhibited, IFN-γ–activated FLAMs. Our GSEA results identified TNF and IFN-β, which were previously associated with modulating costimulatory marker expression (22, 39). With our RNA-seq dataset, we found that in iBMDMs, TNF was expressed following IFN-γ activation regardless of GSK3α/β inhibition; however, in FLAMs, TNF was highly expressed only following IFN-γ activation and GSK3α/β inhibition (Fig. 6A). IFN-β was not expressed in iBMDMs under any conditions, and high expression of IFN-β was observed only in IFN-γ–activated, GSK3α/β-inhibited FLAMs. To confirm the results from RNA-seq analysis, we examined the production of cytokines using a multiplex Luminex assay of supernatants from resting and IFN-γ–activated iBMDMs and FLAMs with and without GSK3α/β inhibition. In agreement with the transcriptomic studies, TNF and type I IFN were increased only in FLAMs following IFN-γ activation and GSK3α/β inhibition (Fig. 6B). We next confirmed these results in primary AMs by stimulating cells with IFN-γ and/or CHIR99021 and quantifying secreted TNF and IFN-β1 by ELISA (Fig. 6C). Together, these data show that inhibition of GSK3α/β in IFN-γ–activated FLAMs results in increased expression of costimulatory molecule-modulating cytokines.

We next tested the sufficiency of either TNF or IFN-β to drive costimulatory marker expression on IFN-γ–activated FLAMs. Resting or IFN-γ–activated FLAMs were treated with recombinant TNF or IFN-β, and surface levels of CD40 were quantified with flow cytometry. While TNF alone did not increase CD40 expression on resting FLAMs, treatment of IFN-γ–activated FLAMs with TNF resulted in a synergistic increase in CD40 expression (Fig. 6D). The addition of type I IFN significantly increased CD40 expression in all conditions, and combination treatment with IFN-γ and IFN-β resulted in higher CD40 expression than treatment with IFN-β alone (Fig. 6E). These data suggest that both IFN-β and TNF synergize with the activation of FLAMs by IFN-γ to increase CD40 expression.

Next, we tested whether the production of either TNF or IFN-β was required for enhanced costimulatory marker expression on GSK3α/β-inhibited, IFN-γ–activated FLAMs. To block the function of IFN-β and TNF, we isolated FLAMs from IFNAR^−/−^ mice and used a TNFR-neutralizing Ab, enabling the role of both cytokines to be tested simultaneously. Resting and IFN-γ–activated wild-type and IFNAR^−/−^ FLAMs in the presence and absence of CHIR99021 and/or anti-TNFR Abs were analyzed for CD40 expression by flow cytometry. We observed that TNF signaling blockade led to a minimal decrease in CD40 expression in IFN-γ–activated, GSK3α/β-inhibited FLAMs, while IFN-β signaling blockade dramatically reduced CD40 expression (Fig. 6F). When TNF was blocked in IFNAR^−/−^ FLAMs, limited changes in CD40 surface expression were observed. Taken together, these data suggest that IFN-β is the primary contributor to the increase in costimulatory marker expression seen in IFN-γ–activated, GSK3α/β-inhibited FLAMs.

### Inhibition of GSK3α/β following *IFN-γ* activation of FLAMs and AMs drives CD4^+^ T cell activation

**FIGURE 7.**
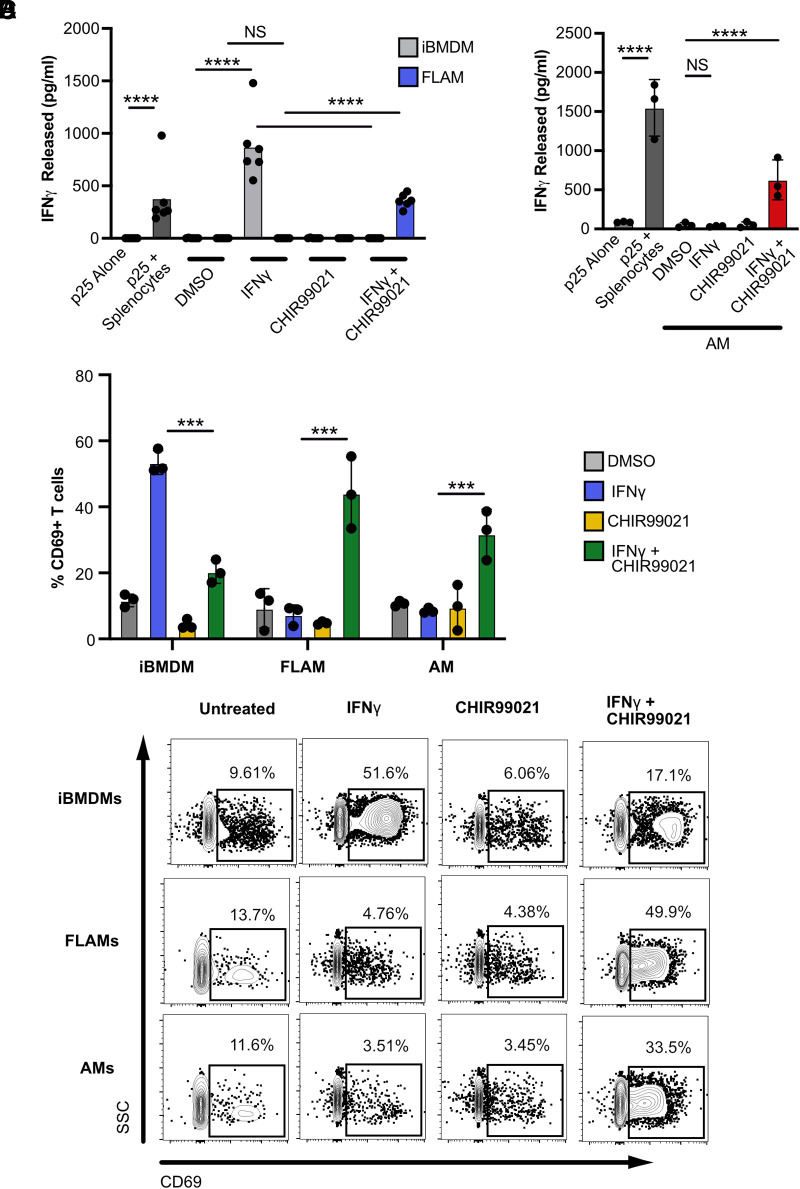
GSK3α/β restrain the ability of AMs to activate CD4^+^ T cells following IFN-γ activation. (**A**) p25 peptide-pulsed iBMDMs and FLAMs treated with DMSO (gray), DMSO and IFN-γ (6.25 ng/ml) (blue), CHIR99021 (10 μM) (yellow), or CHIR99021 and IFN-γ (10 μM and 6.25 ng/ml) (green) for 24 h were cocultured with naive p25-specific T cells for 4 d, and IFN-γ released into the supernatant was quantified by ELISA. P25 T cells alone or P25 T cells with peptide-pulsed splenocytes were used as positive and negative controls for the assay. (**B**) p25 peptide-pulsed AMs treated with DMSO (gray), DMSO and IFN-γ (6.25 ng/ml) (blue), CHIR99021 (10 μM) (yellow), or CHIR99021 and IFN-γ (10 μM and 6.25 ng/ml) (green) for 24 h were cocultured with naive p25-specific T cells for 4 d, and IFN-γ released into the supernatant was quantified by ELISA. Statistical significance was determined with two-way ANOVA and a Tukey test for multiple comparisons. (**C**) p25 peptide-pulsed iBMDMs, FLAMs, and AMs treated with DMSO (gray), DMSO and IFN-γ (6.25 ng/ml; blue), CHIR99021 (10 μM; yellow), or CHIR99021 and IFN-γ (10 μM and 6.25 ng/ml, respectively; green) for 24 h were cocultured with naive p25-specific T cells for 3 d, and the surface expression of CD69 was quantified by flow cytometry. The mean percentage of p25 CD4^+^ T cells expressing CD69 is shown in each cell type from each indicated condition. (**D**) Representative flow cytometry plots. Statistical significance was determined with two-way ANOVA and a Tukey test for multiple comparisons. Cytokine quantification points each represent one biological replicate ± the SD from one representative experiment of two. ****p < 0.0001, ***p < 0.001, **p < 0.01, *p < 0.05. SSC, side scatter.

Costimulatory marker expression is necessary to activate the adaptive immune response during infection (40). Our previous studies found that the increase in Ag presentation and costimulatory markers following IFN-γ activation of BMDMs is sufficient to activate CD4^+^ T cells (14, 20). Given that costimulatory marker expression was not induced in AMs or FLAMs with IFN-γ alone but only in combination with GSK3α/β inhibition, we hypothesized that IFN-γ–activated AMs or FLAMs would not robustly activate CD4+ T cells, whereas IFN-γ–activated, GSK3α/β-inhibited cells would. To test this hypothesis, we used a previously optimized coculture assay with macrophages and TCR-transgenic CD4^+^ T cells that are specific for the *Mycobacterium tuberculosis* peptide p25 and assessed T cell activation in various conditions based on the production of IFN-γ (41, 42). We found that p25 CD4^+^ T cells alone produced no IFN-γ, whereas coculture with peptide-pulsed splenocytes resulted in robust IFN-γ production (Fig. 7A). As expected, p25 CD4^+^ T cells cocultured with resting or CHIR99021-treated iBMDMs or FLAMs did not produce IFN-γ. p25 CD4^+^ T cells cocultured with IFN-γ–activated iBMDMs produced IFN-γ, whereas GSK3α/β blockade in IFN-γ–activated iBMDMs prevented p25 CD4^+^ T cell activation. In FLAMs, IFN-γ activation alone was insufficient to activate p25 CD4^+^ T cells during coculture. In contrast, GSK3α/β inhibition in IFN-γ–-activated FLAMs resulted in the robust production of IFN-γ by p25 CD4^+^ T cells. Similar results were observed when this experiment was repeated with primary AMs (Fig. 7B). p25 CD4^+^ T cells were only activated upon coculture with AMs that were IFN-γ–activated with GSK3α/β inhibition. To examine T cell activation with an orthologous approach, we repeated the above experiment and examined the surface expression of the activation marker CD69 on p25 CD4^+^ T cells. Our results align with the IFN-γ ELISA showing that IFN-γ–stimulated iBMDMs robustly induced CD69 expression on p25 CD4^+^ T cells, while both IFN-γ and GSK3α/β blockade were required for FLAMs and AMs to activate CD69 surface expression (Fig. 7C, 7D). Taken together, these data suggest that distinct macrophage populations have different abilities to directly activate T cell responses and highlight that AMs are restrained in their capacity to directly activate adaptive immune responses.

## Discussion

While AMs are essential for lung function, experimental limitations have prevented a mechanistic understanding of how they uniquely respond to inflammatory signals. Here, we used FLAMs as an ex vivo model of AMs to globally understand transcriptional and functional differences between resident and recruited lung macrophages (23). By comparing the transcriptome of iBMDMs and FLAMs to previously published datasets from ImmGen on various myeloid-derived cells, we found strong similarity between FLAMs and AMs but not between AMs and BMDMs (29). Examination of pathways associated with FLAMs and AMs identified signatures previously associated with AMs, including activation of Ppar signaling, unsaturated fatty acid synthesis, lipid metabolism, and lysosome and peroxisome pathways as defined by gene sets within GSEA (25, 29, 42–47). We also confirmed the expression of AM-associated surface markers including CD11a and Siglec1 in FLAMs, which were expressed at low levels on iBMDMs. Thus, we have shown that FLAMs are a useful model to begin dissecting mechanisms regulating AM maintenance and function ex vivo.

We used FLAMs to dissect how lung macrophages respond to the proinflammatory cytokine IFN-γ. IFN-γ is an important regulator of immunity in the lungs and activates a range of ISGs that drive adaptive immunity, cell autonomous effectors, and cytokines/chemokines (13, 48). Interestingly, our transcriptional profiling found that while both iBMDMs and FLAMs are responsive to IFN-γ stimulation, they induce distinct transcriptional changes following activation. Given the metabolic differences between FLAMs and iBMDMs, our current model suggests that baseline metabolism and differences in IFN-γ–mediated shifts in metabolism drive distinct responses to IFN-γ. Previous studies in BMDMs showed that IFN-γ activation drives a shift in cells toward aerobic glycolysis that is dependent on the activation of HIF1α (19). However, both aerobic glycolysis and oxidative phosphorylation are known to contribute to IFN-γ responses (22). Whether HIF1α plays a role in the different IFN-γ responses between BMDMs and AMs and how metabolism shifts in AMs following IFN-γ activation will be directly examined in the future. Additionally, it is possible that specific transcription factors activated by GM-CSF, such as STAT3 or STAT5, may contribute to the observed phenotypes. By coupling genetic approaches to remove single transcription factors with metabolic flux approaches including Seahorse assays, we will be well positioned to understand these mechanisms driving the interlinked metabolic and transcriptional responses following AM activation with IFN-γ.

The metabolism-associated kinases GSK3α/β were previously associated with macrophage functions including IFN-γ activation in iBMDMs (49, 50). Thus, we examined the role of GSK3α/β in FLAMs. In contrast to iBMDMs, we found that GSK3α/β do not control IFN-γ–dependent MHC-II upregulation in FLAMs or AMs, and our transcriptional profiling showed that there are macrophage subset-specific roles for GSK3α/β in regulating the IFN-γ response. Unlike in iBMDMs, the inhibition of GSK3α/β in combination with IFN-γ activation in FLAMs resulted in a dramatic shift in the transcriptional landscape. It was beyond what was seen with either GSK3α/β inhibition or IFN-γ activation alone. What drives the synergistic response of AMs to both IFN-γ and GSK3α/β inhibition remains an open question. One potential explanation for this synergy is the observation that type I IFN responses are robustly induced only in IFN-γ–activated FLAMs with GSK3 inhibition. Type I IFNs can be induced by endogenous ligands from the mitochondria, such as mitochondrial DNA or RNA, as well as changes in cholesterol metabolism (51–53). The contribution of these distinct IFN pathways in IFN-γ–activated GSK3α/β-inhibited FLAMs and whether type I IFNs drive the observed transcriptional changes will need to be tested in the future. Altogether, our results show that GSK3α/β are important regulators of IFN-γ responses in AMs that differ from those in BMDMs.

Throughout our study, we noticed major differences in the regulation of T cell modulatory markers between iBMDMs and FLAMs. While costimulatory molecules were robustly induced in iBMDMs with IFN-γ activation alone, these molecules were only induced in FLAMs if GSK3α/β were also inhibited. These differences in MHC-II and costimulatory molecule expression had functional implications, because FLAMs and AMs only activated CD4^+^ T cells during IFN-γ activation if GSK3 was also blocked. Our data support different roles for various macrophage subtypes in directly modulating the adaptive immune system. Previous studies suggest that AMs are not efficient activators of naive T cells (10, 11). In fact, robust activation of T cells by AMs was associated with worse clinical outcomes during infection with SARS-CoV-2 in a manner that was dependent on both IFN-γ and TNF (35). We speculate that AMs respond to IFN-γ in a way that prevents overly robust activation of T cells and limits deleterious lung damage. However, when combined with other inflammatory signals, including type I IFN or TNF, IFN-γ drives AMs to robustly activate T cell responses. It is possible that pathogens such as *M. tuberculosis* take advantage of the restrained T cell–activating capacity of AMs to prevent detection and initiate lung infections, but this hypothesis needs to be directly tested.

The strength of the approach presented here lies in the ease and tractability of using FLAMs as a model of AMs to identify mechanisms that make AMs unique. Throughout our study, significant differences were observed between iBMDMs and FLAMs in gene expression, surface protein expression, innate immune pathway activation, and the functional capacity to activate CD4^+^ T cells. Our data suggest an urgent need to define AM-specific regulation of host responses rather than extrapolating immune pathway regulatory mechanisms previously observed in BMDMs. Filling this gap may identify potential targets for lung-specific therapies and provide a better understanding of how AMs initiate immune responses while maintaining pulmonary function. It will also be important to directly dissect these pathways within the lung environment, which is a limitation of our current study. While the ex vivo propagation of FLAMs may introduce conditions not seen in vivo, our experiments with primary AMs suggest that this is not due to major differences in cell function. However, cell type–specific knockout animals will be required to define the role of GSK3α/β in distinct macrophage subtypes. Work is underway to generate both GSK3α and/or GSK3β conditional knockout animals in distinct macrophage populations using LysM- and CD11c-driven Cre recombinase. These mice will enable future work to better define the role of GSK3α/β directly in lungs and how they contribute to AM function.

In conclusion, this study shows that FLAMs are a useful model to interrogate mechanisms that make AMs unique among macrophage subsets. IFN-γ responses are differentially regulated in AMs and BMDMs, and GSK3α/β modulates inflammation and T cell activation by AMs. Together, these findings build upon previous studies that suggest that there are key mechanistic differences between AMs and BMDMs and provide tools to better understand these differences and their roles in maintaining pulmonary function in health and disease.

## Supplementary Material

Supplemental Table 1 (XLS)

Supplemental Table 2 (XLS)

Supplemental Table 3 (XLS)

Supplemental Table 4 (XLS)
